# Cellular, Molecular, and Genetic Mechanisms of Avian Beak Development and Evolution

**DOI:** 10.1146/annurev-genet-111523-101929

**Published:** 2024-11-14

**Authors:** Richard A. Schneider

**Affiliations:** Department of Orthopaedic Surgery, University of California at San Francisco, San Francisco, California, USA

**Keywords:** cranial neural crest, species-specific size and shape, craniofacial development, avian beak, quail–duck chimeras, quck, evolutionary-developmental biology

## Abstract

Diverse research programs employing complementary strategies have been uncovering cellular, molecular, and genetic mechanisms essential to avian beak development and evolution. In reviewing these discoveries, I offer an interdisciplinary perspective on bird beaks that spans their derivation from jaws of dinosaurian reptiles, their anatomical and ecological diversification across major taxonomic groups, their common embryonic origins, their intrinsic patterning processes, and their structural integration. I describe how descriptive and experimental approaches, including gene expression and cell lineage analyses, tissue recombinations, surgical transplants, gain- and loss-of-function methods, geometric morphometrics, comparative genomics, and genome-wide association studies, have identified key constituent parts and putative genes regulating beak morphogenesis and evolution. I focus throughout on neural crest mesenchyme, which generates the beak skeleton and other components, and describe how these embryonic progenitor cells mediate species-specific pattern and link form and function as revealed by 20 years of research using chimeras between quail and duck embryos.

## 1. INTRODUCTION

Bird beaks are remarkable for their range of morphological diversity. Beak size and shape often evolve rapidly to support ecological, behavioral, and functional requirements in astonishing ways. Most studies, starting with those by Charles Darwin on Galápagos finches (Passeriformes) and domestic pigeons (Columbiformes) (26, 27), have illuminated the role of natural and artificial selection in driving beak adaptations at the population level. By contrast, developmental mechanisms through which avian beaks acquire their species-specific anatomy have been less well understood. Recent work in this area, however, has begun to shed light on where, when, and how modifications at the cellular, molecular, and genetic levels can generate species-specific pattern and the phenotypic variation necessary for beak evolution.

In this review, I detail cellular, molecular, and genetic mechanisms underlying avian beak development and evolution. I start with a concise summary of the phylogenetic origins of birds and their beaks from dinosaurian reptiles. I then overview beak anatomy and provide examples of the many ways that beaks have diverged morphologically across avian taxa, particularly in the context of feeding strategies and other ecological adaptations. To emphasize what all bird beaks share in terms of their developmental anatomy, I describe the common embryonic origins of the primordia, tissues, and cells that form the avian beak. For this, I concentrate on what has been learned through fate mapping and other cell lineage analyses, tissue recombination and transplant experiments, and gain- and loss-of-function approaches about how each constituent part mediates beak patterning. I give special attention to the effects of signaling molecules secreted by epithelial tissues on the specification of axes, identity of individual components, and outgrowth of the beak. I also delve deeply into the role of neural crest mesenchyme (NCM), which is an embryonic precursor population that builds the beak skeleton, in mediating species-specific pattern and integrating form and function. Much of what we know on the latter topic comes from work in my lab using embryos of Japanese quail (*Coturnix japonica*) and White Pekin duck (*Anas platyrhynchos domesticus*), which I offer as an illustration of how molecular and cellular programs for beak patterning can be differentially regulated to generate evolutionary changes in morphology. Subsequently, I explore genetic and epigenetic mechanisms of beak evolution as elucidated through comparative genomics across various bird taxa as well as genome-wide association studies (GWAS) that focus on beak size and shape. I end my review with a brief discussion of how what we have learned about beak patterning in birds thus far portends potential future directions for the field.

## 2. EVOLUTIONARY ORIGINS AND FUNCTIONAL ANATOMY OF THE AVIAN BEAK

The toothless beak or bill is a defining feature of modern birds. While beaks evolved independently in multiple extinct reptile clades and are also present in extant turtles, birds demonstrate the greatest amount of beak diversity, are considered the most successful group of beaked amniotes, and may have survived mass extinction in part because their edentulous beaks could help support a granivorous diet (76, 82, 148). Avians first arose as a lineage from small coelurosaurian theropods, and these early bird-like dinosaurs, including the famous *Archaeopteryx* that lived approximately 150 million years ago during the Late Jurassic, had typical reptilian upper and lower jaw skeletons complete with rows of uniform teeth (100). From around 85 to 65 million years ago in the Late Cretaceous, these reptilian teeth were reduced, birds went from being toothed to toothless, and jaws evolved into beaks and bills (28, 39).

**Figure 1:**
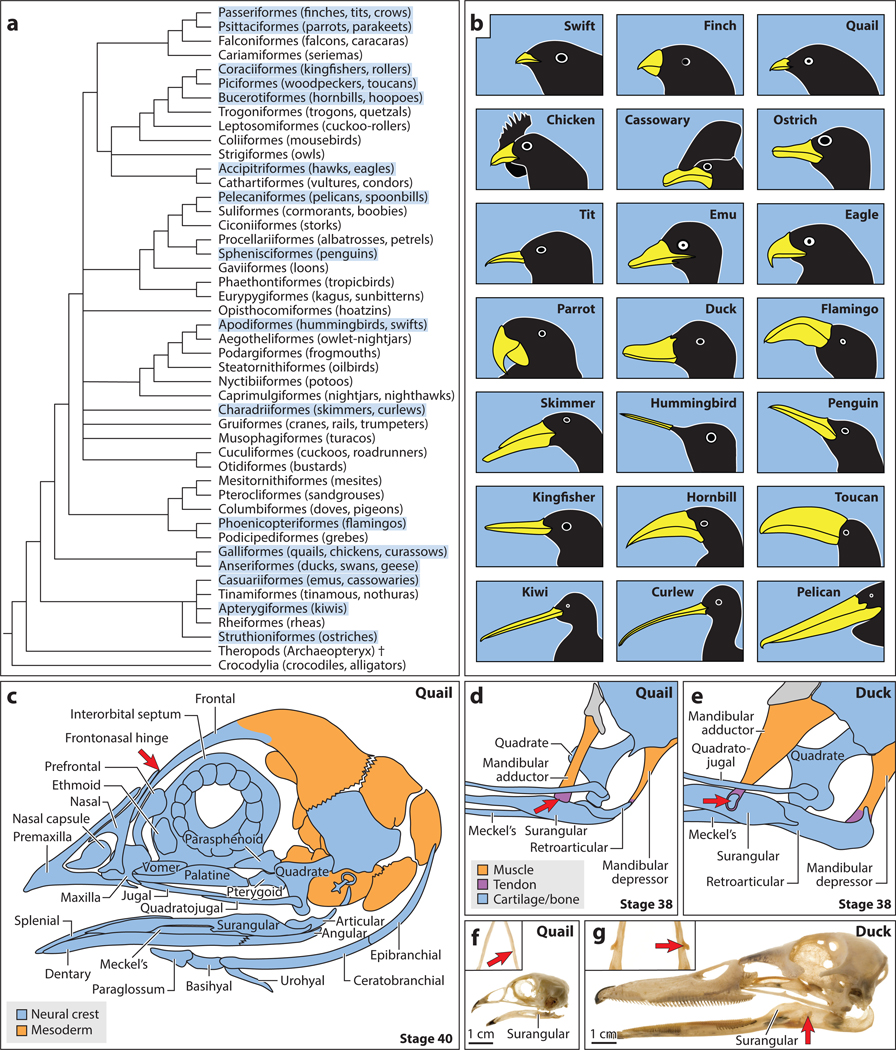
Evolution, anatomy, and functional morphology of bird beaks. (*a*) Hypothetical relationships among orders of birds, based on References 13, 17, 80, and 106. Relationships among many clades remain unresolved. The dagger indicates an extinct taxon. Beaks from orders highlighted in blue are represented in panel *b*. (*b*) Diversity of bird beak size and shape. Bird drawings are not to scale. (*c*) Bones and cartilages of the embryonic bird beak skeleton are derived from either the neural crest (*blue*) or mesodermal (*orange*) mesenchyme. A kinetic hinge (*red arrow*) forms between the frontal and nasal bones. Panel *c* adapted from Reference 98. (*d*) The jaw closes via the contraction of mandibular adductor muscles and opens via the contraction of mandibular depressor muscles (*orange*). Feeding by pecking in quail correlates with a short lever arm (i.e., retroarticular process) and relatively small mandibular adductor and depressor muscles. The tendon (*purple*) of the mandibular adductor muscle of quail inserts dorsally (*red arrow*) on the surangular bone (*blue*). (*e*) In duck, rapid acceleration of the jaw during feeding is achieved by greater force and relatively larger mandibular adductor and depressor muscles, a longer lever arm of the retroarticular process, and the formation of secondary cartilage (*red arrow*) within the tendon enthesis that inserts laterally on the surangular bone. Panels *d* and *e* adapted from Reference 131. (*f*) Adult quail and (*g*) duck head skeletons showing differences in functional morphology, especially along the surangular bone where quail have a slight bony ridge (*red arrow*) and duck have a robust protruding coronoid process (*red arrows*). Panels *f* and *g* adapted from Reference 144.

Historically, tooth loss in birds was thought to have evolved as an adaptation for specialized feeding and weight reduction for flight, but some researchers have recently argued that the principal selective pressure for edentulism in avian lineages may have been for more rapid rates of embryonic growth, as opposed to the substantially slower reptilian incubation times that likely constrained toothed dinosaurs (38, 148). After mass extinctions terminated their nonavian dinosaurian relatives and over the past 65 million years, modern birds radiated dramatically into more than 10,000 species worldwide (17, 23). There are approximately 45 recognized orders of extant birds (Figure 1a), but their relationships to one another and which birds belong to each clade remain controversial (13, 65, 66, 80, 106, 133). While modern birds persist as dinosaurian descendants with many reptilian traits, their beaks have become independently derived in important ways that are characteristic of birds, especially the underlying skeletal anatomy and elaboration of the rhamphotheca, which is the keratinized outer horny sheath that covers the beak skeleton (39, 84).

Rhamphothecae and their many keratinized elaborations, including lamellae, ridges, hooked tips, serrations, and crushing plates, contribute to overall beak function by facilitating tasks such as filter feeding in ducks (Anseriformes) and flamingos (Phoenicopteriformes), nutcracking in finches (Passeriformes) and parrots (Psittaciformes), piercing and tearing in eagles (Accipitriformes) and kingfishers (Coraciiformes), digging and probing in curlews (Charadriiformes) and kiwis (Apterygiformes), and pecking and grasping in penguins (Sphenisciformes) and quail (Galliformes) (39, 63, 154) (Figure 1b). Additionally, rhamphothecae can be colored in various patterns as part of the ornamentation that birds deploy for social displays, communication, and sexual selection. Such color can arise through concurrent mechanisms including (*a*) structural coloration, which involves photonic nanostructures in epidermis that cause iridescence as well as ultraviolet, dark blue, light blue, green, and yellow hues (114); (*b*) consumption of dietary carotenoids in plants and insects and subsequent deposition of red, yellow, and orange pigments in the beak, which is mediated by genes such as *Beta-carotene oxygenase 2* (*Bco2*) (36); and/or (*c*) synthesis by NCM-derived melanocytes of brownish-black eumelanin and reddish pheomelanin that are secreted directly into epidermis and can be alternated to generate discrete pigment patterns (35).

The size and shape of rhamphothecae can also vary due to changes in the underlying facial skeleton and elaboration of individual bones. Some birds, such as cassowaries (Casuariiformes) and hornbills (Bucerotiformes), form large bulbous casques that extend from their beaks along the top of their heads, whereas other birds, such as toucans (Piciformes), can have greatly expanded rostra (Figure 1b), or there can be projections from the lower beak as in curassows (Galliformes) (154). Among the most massive are the large, broad, spatula-like beaks of spoonbills, pelicans, and boat-billed herons (Pelecaniformes) (39). Such elaborations are usually supported by light and spongy pneumatized bone that is integrated with other bones in the beak skeleton. The upper portion of the beak skeleton (from distal to proximal) consists of the premaxillary, nasal, maxillary, vomer, jugal, palatine, and pterygoid bones (Figure 1c), most of which are paired and fuse in adults into a single mass that articulates at a kinetic hinge between the nasal and frontal bones (8, 154). Movement of the upper beak relative to the rest of the braincase helps birds use their beaks much like hands for grasping different kinds of items, especially since their forelimbs have become wings dedicated to flight (5, 6), and some birds, such as parrots (Psittaciformes), have evolved additional adaptations in their craniofacial hinge to sustain specific modes of feeding and locomotion (11, 135, 149). Although cranial kinesis is not unique to birds (128), the ability of birds to move their beak upwards and downwards relative to the braincase facilitates various kinds of functional enhancements, such as increased range of motion, rapid closure, wider gape, and fine motor control, thus enabling highly specialized behaviors for prey capture, tool use, foraging, nest building, communication, courtship, grooming, and feeding, among many others (39, 41, 103, 104, 124, 154).

The extreme degree of mobility in bird beaks is due to multiple points of articulation with the remaining head skeleton, which in palaeognathous birds such as ostriches (Struthioniformes) and emus (Casuariiformes) resembles a basic four-bar linkage system (5, 8, 56). A thin, rod-like quadratojugal bone (Figure 1c) typically connects the posterior end of the upper beak to the ventrolateral aspect of the quadrate bone, which forms a highly movable joint. The quadrate further articulates with the braincase along its dorsal surface, the pterygoid bone of the palatal region on its anterior margin, and the articular bone of the lower jaw on its ventral side (154) (Figure 1c–e). The lower jaw also contributes to the beak skeleton and, in addition to two articular bones, consists of five other paired bones often fused into two rod-like mandibles around Meckel’s cartilage. These elements are the dentary and surangular on the lateral side, and splenial and angular on the medial surface (4) (Figure 1c). At their distal tips, the mandibles are joined along the midline into a solid immovable symphysis that usually opposes the distal tip of the upper beak. In some birds, however, there are striking differences in how the upper and lower tips of the beak approximate one another, such as the kea (*Nestor notabilis*), which has a shorter lower beak, and black skimmer (*Rynchops niger*), which has a longer lower beak relative to the upper beak (31) (Figure 1b). Notably, when black skimmers hatch, their upper and lower jaws are about the same length, but by the time they fledge at four weeks, their lower jaw is almost a centimeter longer than their upper jaw. Such beak architecture equips these birds with an uncanny ability to skim fish and assorted small prey items near the water’s surface. Another example includes red crossbills (*Loxia curvirostra*), whose asymmetrical upper and lower beaks traverse one another at the distal tips, which enables these birds to pry open conifer cones and extract seeds (144). Such developmental and evolutionary variation indicates that upper and lower beak lengths are regulated through independent genetic, molecular, and cellular mechanisms that remodel the beak at late embryonic and posthatching stages of growth.

The rapid evolutionary elongation of the upper portion of the beak, even in some nonavian theropods, such as the Late Cretaceous oviraptorosaur *Citipati* (42), coincides with the extended growth and fusion of the premaxillary bone during development (6), which becomes conspicuously enlarged and a vital component of the kinetic system that enables the upper jaw to move up and down independent of the braincase (5). Furthermore, upper beak length in birds is almost exclusively driven by expansion of the premaxillary bones, while maxillary bones become highly reduced; this is distinct from mammals and other nonavian amniotes in which maxillary bones are the primary driver of rostral elongation (7, 150). Lower jaw length is established through separate mechanisms (22, 118). Overall, the extent to which beak size and shape have varied during evolution appears constrained internally by developmental and anatomical modules in the skull that are highly integrated and shared among most lineages of birds (40, 50, 89, 93, 151). Moreover, rates of change and adaptive radiations of the beak appear relatively stable at different taxonomic levels and time scales (23, 55), which may be due to inherent tension between modularity within the developing skull and selective pressures from dietary ecology (14, 94, 99).

Species-specific evolution has also occurred in other skeletal elements as well as soft tissues, such as muscles and tendons, associated with upper and lower portions of the beak. For example, within the oral cavity of the beak lies the lingual apparatus and its supporting hyoid skeleton, which consists of the paraglossum that inserts into the tongue, basihyal and urohyal elements along the midline, and paired horns of the hyoid made up of ceratobranchials and epibranchials (4, 154) (Figure 1c). In the absence of teeth and as an essential component of beak function that supports capture, manipulation, filtering, processing, sucking, drinking, and swallowing of food items, the tongue and hyoid apparatus have undergone tremendous morphological diversification across birds. This is evidenced most vividly in woodpeckers (Piciformes) and hummingbirds (Apodiformes), which have evolved distinctive tongue and hyoid adaptations, such as extreme elongation in association with insectivorous and nectarivorous diets (37, 68, 109, 154).

The complex movements of the avian beak, chiefly regarding kinesis, are capacitated by a series of ligaments that extend between bones or parts of bones, run along articulations, or bridge over articulations to function as linkage ligaments spanning several bony units or parts of bony units (8, 71). Also, an intricate and somewhat redundant arrangement of jaw muscles works in partnership with linkage ligaments, tendons, and other muscle connective tissues to allow birds to combine myriad beak movements as part of their specialized behaviors. Although there are numerous examples of individual jaw muscles becoming highly specialized, lost, or newly gained in different lineages, such as parrots (Psittaciformes) (135, 136), the generalized arrangement in most birds consists of seven pairs of muscles, including muscles that open the jaws (i.e., mandibular protractors and depressors), simultaneously close the upper and lower jaws (i.e., pterygoids and the pseudotemporalis), and lift and close the lower jaw (i.e., mandibular adductors) (4, 71) (Figure 1d,e). The ability of the beak to perform and support the lifestyle of any given bird is powered by the structural and functional integration of jaw muscles with hinges, articulations, ligaments, tendons, bones, and cartilages during development (154). The coevolution of components within the beak musculoskeletal system becomes most apparent when comparing divergent species with distinct feeding mechanisms.

Waterfowl such as duck (Anseriformes), for instance, utilize their broad bills to dredge sediment for food items. To add leverage to this specialized feeding behavior, mandibular adductor muscles attach along the proximal portion of the lower jaw and laterally to a large protruding coronoid process on the surangular bone (131). The coronoid process endows duck with a robust insertion site for transmitting high-magnitude forces that contribute to their suction pump and levered-straining jaw movements (143). The duck coronoid process, which forms from secondary cartilage at the tendon insertion (i.e., enthesis), requires mechanical stimulation for its induction and maintenance (131). By contrast, quail and chick (Galliformes), which peck at their food and use the distal tips of their beaks more like a pair of forceps, have mandibular adductor muscles that insert dorsally toward the midpoint of the lower jaw; these birds lack secondary cartilage on their coronoid process, which instead appears as a slight bony ridge (Figure 1f,g). Insights on how such differences in form and function arise during evolution have been gained by comparing molecular, cellular, and biomechanical mechanisms that operate during beak development in divergent lineages of birds (72, 131, 143). As described below, we have been implementing this strategy using quail and duck embryos because their lineages are separated by over 100 million years of evolution (102) and they show clear species-specific differences in beak functional morphology associated with specialized feeding behaviors (144).

## 3. EMBRYOLOGY OF THE AVIAN BEAK

**Figure 2:**
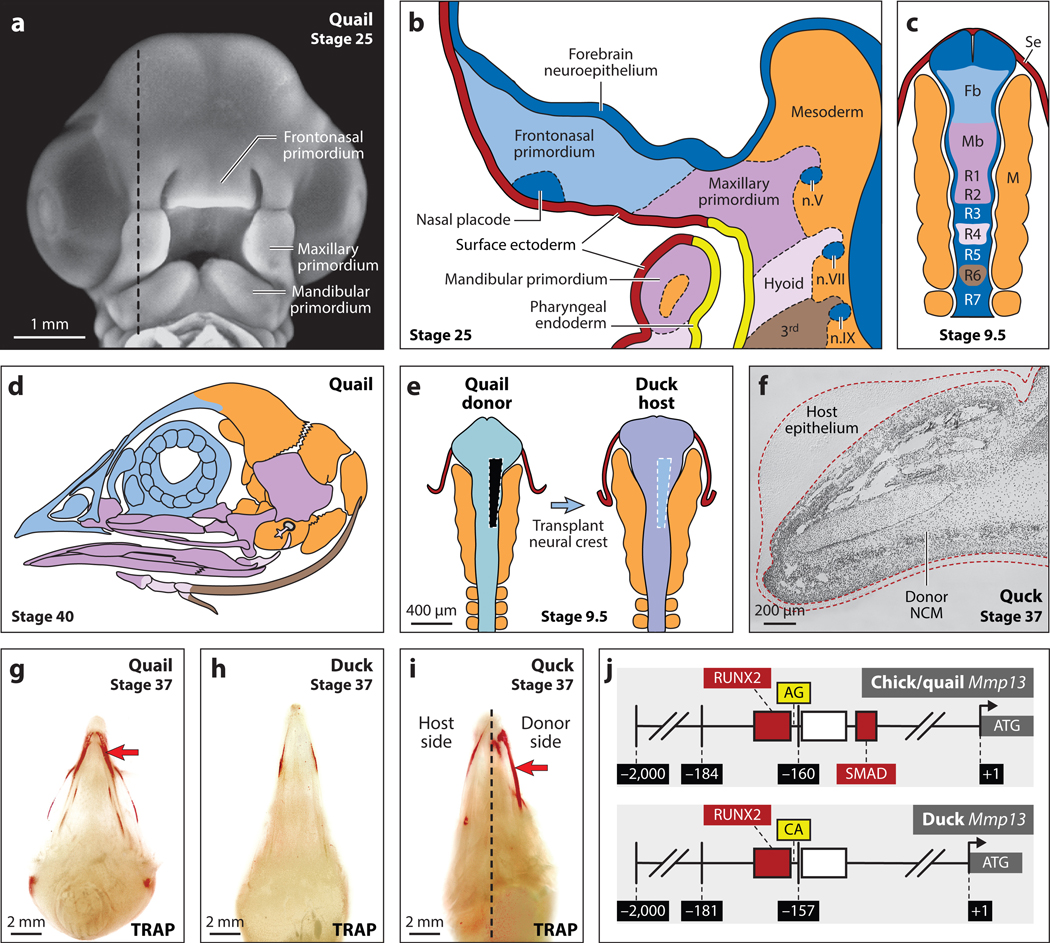
Embryology and patterning of bird beaks. (*a*) Quail frontonasal, maxillary, and mandibular primordia in frontal view. The black dashed line represents the section plane in panel *b*. (*b*) Sagittal view of frontonasal, maxillary, and mandibular primordia, as well as the hyoid and 3^rd^ pharyngeal arches with surface ectoderm (*red*), pharyngeal endoderm (*yellow*), and forebrain neuroepithelium (*dark blue*). Beak primordia contain neural crest mesenchyme (NCM), nasal placode (*dark blue*), cranial nerve ganglia (n.V, n.VII, n.IX; *dark blue*), and mesoderm (*orange*). (*c*) Dorsal view of NCM from the posterior forebrain (Fb) and anterior midbrain (Mb), which produce frontonasal structures (*light blue*), and from posterior midbrain and hindbrain rhombomeres (R), which make maxillary and mandibular derivatives (*purple*). Hyoid arch forms from R4 (*pink*), and the 3^rd^ arch contains NCM from R6 (*brown*). NCM migrates through mesoderm (M; *orange*) and under surface ectoderm (Se; *red*). (*d*) NCM generates the upper and lower portions of the beak skeleton in a manner that corresponds to their embryonic distribution in the frontonasal primordium (*light blue*), maxillary and mandibular primordia (*purple*), hyoid arch (*pink*), and 3^rd^ arch (*brown*), whereas mesoderm forms the posterior skull vault and base (*orange*). Panels *a*–*d* adapted from Reference 116. (*e*) NCM is transplanted unilaterally from quail to duck to make chimeric quck. Panel *e* adapted from Reference 33. (*f*) Quail donor NCM (*black dots*) is observed in the beak, whereas the duck host is unlabeled. The quck egg tooth is quail-like despite arising from the duck host (*red dashed line*). (*g*) Staining for bone resorption [tartrate-resistant acid phosphatase (TRAP)] in quail versus (*h*) duck shows species-specific differences in activity and spatial domains (*red arrow*). (*i*) Quck reveal that NCM controls bone resorption as indicated by higher quail-like levels of TRAP activity on the donor side (*red arrow*). Panels *g*–*i* adapted from Reference 31. (*j*) Chick/quail and duck *Mmp13* promoters upstream from the translational start site (ATG). Chick/quail contain a SMAD-binding element and two single-nucleotide polymorphisms (SNPs) (AG) near a RUNX2-binding element, whereas duck lack the SMAD-binding element and have CA; these polymorphisms account for species-specific expression of *Mmp13* during beak patterning. Panel *j* adapted from Reference 130.

Despite tremendous variation in adult beak size and shape across birds, at early embryonic stages all bird beaks arise from equivalent facial primordia, tissues, and cells. The upper aspect of the beak is formed from frontonasal and paired maxillary primordia, while the lower portion develops from paired mandibular primordia (Figure 2a). These primordia consist of epithelial and mesenchymal cells that originate from three embryonic germ layers (i.e., ectoderm, mesoderm, and endoderm) and play distinct structural roles during beak development. Epithelia are organized as polarized sheets of tightly connected cells, and those associated with the beak include the endoderm-derived lining of the pharynx and ectoderm-derived neural tube. The beak also contains epithelial tissues made from surface (i.e., nonneural) ectoderm that forms the lining of the oral cavity and epidermis (116) (Figure 2b), as well as placodal ectoderm that produces nasal epithelium. Epidermis becomes stratified into discrete layers, and the upper layer is composed of the nonliving stratum corneum, which makes up the keratinized rhamphotheca and its associated egg tooth (84). A thin basement membrane separates epidermis from underlying dermis, which is mesenchymal in origin.

Mesenchyme, which is defined as loosely associated stellate-shaped cells, arises in the beak from both the neural crest and mesoderm (98). NCM that originates within the dorsal margins of the neural tube during neurulation migrates into the facial primordia (62). Specifically, NCM from the posterior forebrain and anterior midbrain populates the frontonasal primordium, whereas NCM from the posterior midbrain and anterior hindbrain migrates into the maxillary and mandibular primordia (121) (Figure 2b,c). These fate maps are primarily based on lineage-tracing studies using quail–chick chimeras and retroviral labeling, which have also demonstrated that NCM gives rise to a variety of beak tissues, including all bone, cartilage, dermis, and muscle connective tissues such as ligaments and tendons (24, 98, 115). NCM also makes sensory neurons and glia, Schwann cells, pericytes, and pigment-producing melanocytes that infiltrate epidermis and become the source of black and brown color in the rhamphotheca. Mesodermal mesenchyme in the beak originates primarily from paraxial and lateral plate mesoderm and gives rise to vascular endothelium, osteoclasts, and voluntary muscles that open and close the beak and support its kinesis (24, 98).

In response to reciprocal signaling between epithelium and mesenchyme, the facial primordia expand through a highly orchestrated process, contact one another along the midline and at their lateral margins, fuse such that mesenchyme becomes confluent, and ultimately generate upper and lower bones and cartilages of the beak skeleton. In particular, premaxillary, nasal, ethmoid, prefrontal, and frontal bones differentiate around the cartilaginous nasal capsule and interorbital septum within the frontonasal primordium; maxillary, vomer, palatine, parasphenoid, pterygoid, jugal, and quadratojugal bones as well as quadrate cartilage arise in the maxillary primordia; and dentary, splenial, surangular, angular, and articular bones form around Meckel’s cartilage in the mandibular primordia (62) (Figure 2b–d).

Similarly, in most amniotes, complex epithelial–mesenchymal interactions underlie odontogenesis in the oral cavity (67), but, in birds, tooth loss likely occurred through a truncation of reptilian odontogenic programs in early lineages of avian and nonavian theropods that also evolved edentulous beaks (29, 82). Evidence comes from both the fossil record and experiments suggesting that changes to epithelial–mesenchymal interactions promoting keratinization of oral epithelium for rhamphothecae may have inhibited embryonic tooth formation (141). This could have occurred via a positional shift of odontogenic epithelium relative to dental mesenchyme (28), especially since chick embryos seem to retain rudiments of tooth germs as transient epidermal thickenings comparable to dental laminae of mammals and some odontogenic genes are still expressed (20, 74). Moreover, transplanting mouse NCM into chick embryos induces expression of odontogenic genes and begets tooth-like structures, which reflects that oral epithelium in birds may continue to possess components of the odontogenic program (67, 92). Remnants of an ancient odontogenetic program in birds is further evidenced by the appearance of tooth-like evaginations in a naturally occurring chick mutant (59) and by pseudoteeth, which lack tissues of true teeth (i.e., dentin, enamel, and cementum), in some lineages of extinct and extant birds (81, 83).

Although the cell lineages that populate facial primordia and the tissue-level organization of primordia themselves remain highly conserved among birds, species-specific changes in growth trajectories of the primordia appear in early development and can account for later morphological divergence (127, 150). For example, we have found significant differences in the amount of NCM that migrates into mandibular primordia of duck, which have long bills, versus quail, which have short, blunt beaks (48) (Figure 1b,f,g). Duck embryos generate approximately 15% more premigratory NCM along their midbrain and anterior hindbrain, which then migrates into the facial primordia. In fact, the overall midbrain shape during neurulation in duck and other Anseriformes, such as geese, is expanded laterally compared to Galliformes, such as chick and quail (46, 79), and this seems to presage and correlate with a larger allocation of NCM (48, 119) (Figure 2e).

Over time, this augmented progenitor pool expands due to species-specific differences in cell proliferation dynamics and cell cycle length, and duck amass two times as many cells in their mandibular primordia as do quail once they reach a stage where differentiation begins in the lower jaw skeleton. Duck embryos develop at a slower rate than quail embryos (28 days from fertilization to hatching versus 17 days) and cell cycle length in mandibular mesenchyme of duck is longer (13.5 h) than in quail (11 h). By maintaining their slower intrinsic maturation rate over a longer time period, duck embryos progressively increase jaw size during development (47, 48, 118, 119). In this way, duck leverage incubation time as an intrinsic mechanism contributing to beak length, which supports prior observations in birds showing a positive relationship between innate rates of growth and overall body size (132). Yet the extent to which this phenomenon applies to other long-billed birds remains unclear. Multiple developmental mechanisms must regulate beak growth since there appear to be numerous examples of long beaks on small, rapidly developing birds, such as on sword-billed hummingbirds (*Ensifera ensifera*), which have beaks up to 11 cm that are often longer than their bodies (1). Additional mechanisms shown to pattern avian beaks are described below.

## 4. MOLECULAR AND CELLULAR MECHANISMS OF BEAK PATTERNING

During normal development, every beak acquires its characteristic anatomy through a combination of species-generic and species-specific patterning mechanisms (120). Species-generic patterning replicates the basic craniofacial blueprint shared by all jawed vertebrates and includes axial orientation (e.g., dorsoventral, mediolateral, proximodistal, and oral–aboral), anatomical identity (e.g., upper jaw versus lower), and tissue type (e.g., cartilage, bone, muscle, tendon, and nerve). By contrast, species-specific patterning mechanisms superimpose on species-generic pattern those relatively unique morphological traits that distinguish one species (or a higher taxonomic level) from another, and these generally appear finely tuned to functional, ecological, sexual, or other types of selective pressures (120). In this context, and in terms of avian anatomy, species-specific patterning has mostly been focused on measuring and understanding how changes in beak size and shape are acquired during development and evolution (47, 118, 119).

Epithelia surrounding beak primordia are the principal source of signals required for establishing species-generic pattern and for maintaining the proper outgrowth of individual beak components (47). For instance, ectodermally derived epithelium around the frontonasal primordium facilitates proper expansion and orientation of skeletal elements along the dorsoventral, mediolateral, and proximodistal axes. Experimentally rotating frontonasal epithelium can cause mirror image duplications of upper beak structures along the dorsoventral axis (62, 88). Epithelia associated with the nasal placode and floor of the forebrain also help pattern the upper beak (87, 134). Likewise, endodermally derived epithelium that lines pharyngeal portions of the mandibular primordia ensures proper axial orientation, anatomical identity, and growth of cartilage and bone in lower portions of the beak skeleton (24, 52, 61). When endodermal epithelium is surgically rotated or removed, the accompanying NCM-derived skeleton either becomes reoriented or fails to form.

Each of these epithelia impart species-generic pattern by secreting various combinations of ligands from well-characterized pathways, including Bone Morphogenetic Protein (BMP), Transforming Growth Factor Beta (TGFβ), Sonic Hedgehog (SHH), Fibroblast Growth Factor (FGF), and Wingless-Related (WNT), that act on NCM (15, 19, 49, 87, 122, 125, 142). After migrating into the facial primordia and settling alongside epithelia, NCM responds by expressing a diverse set of transcription factors and other genes that affect its anatomical identity (24, 44, 45, 53, 129) and spur its patterned outgrowth (3, 60, 88, 107). Varying levels of molecules expressed by epithelia, such as retinoic acid and the BMP antagonist Noggin, can, for example, transform maxillary primordia into a frontonasal primordium (78) seemingly by redirecting the flow of downstream gene regulatory networks within the responding NCM (97, 107). Similarly, experiments that manipulate endothelin-mediated signaling and expression of downstream effectors in NCM, including the combinatorial code of *Dlx* genes, can affect the axial orientation of the jaw skeleton and transpose the anatomical identity of maxillary and mandibular primordia (30, 69).

Reinforcing the finding that anatomical identity is established locally in response to epithelial signals are classic embryological experiments demonstrating that when midbrain and anterior hindbrain populations of NCM are surgically rotated by 180° (which transposes frontonasal and mandibular NCM precursors), they can still generate normal facial and jaw skeletons appropriate for their new location (98). This result is likely enabled by the fact that NCM from the midbrain and anterior hindbrain does not rely on homeotic selector genes from the *Hox* complex as part of its transcriptional network (98), which may also promote its plasticity and rapid evolvability (77, 113). For example, when *Hoxa2* is overexpressed in NCM destined for maxillary and mandibular primordia, mandibular components of the jaw skeleton take on the identity of the hyoid, which is a more posterior pharyngeal arch (53, 123). But when *Hoxa2* is eliminated in NCM bound for the hyoid arch, either by surgical replacement with non-*Hox*-expressing NCM destined for frontonasal or mandibular primordia or through targeted knockout (i.e., *Hoxa2* mutants), then mandibular skeletal structures form in place of hyoid arch elements (51, 98, 110). *Hoxa2* expression in NCM is dependent on secretion of FGF8 by neuroepithelium at the midbrain–hindbrain boundary and crosstalk with epithelium once NCM has migrated into the pharyngeal arch (24, 138).

Thus, molecules secreted by ectodermally and endodermally derived epithelia function as local sources of signals for species-generic pattern in the beak by eliciting and/or maintaining programmatic responses from large sets of genes that interact via complex regulatory networks in NCM (123, 140). Notably, signals that pattern the axes and impart anatomical identity to the facial primordia appear highly conserved across vertebrates (120) and rather generic in nature since epithelia that express these signals are somewhat interchangeable (57, 87, 108). This degree of conservation indicates that all birds likely deploy the same signaling pathways and gene regulatory networks to specify their axes and establish the anatomical identity of homologous structures from which their beaks get built. Yet this leaves open the questions of where, when, and how species-specific differences in beak morphology arise.

Experiments in birds have revealed that programmatic responses within NCM are species-specific and direct spatial and temporal domains of gene expression in adjacent epithelia (87, 121) through a series of reciprocal, dynamic, hierarchical, and cell-autonomous interactions (24, 98). NCM relies on these interactions to integrate species-specific information about size and shape with species-generic properties while building cartilages and bones of the beak; directing epidermal differentiation, including the egg tooth and color pattern; and guiding associated musculature (47). The extent to which NCM functions as a source of species-specific patterning information and intrinsic mechanisms these cells use to control beak size and shape have been made most apparent through comparative analyses and subsequent experimental manipulation of gene expression, as well as through surgical transplants that generate chimeras, which leverage differences in the functional morphology of the beak among birds (144). These strategies have pinpointed where, when, and how developmental programs become differentially regulated on the molecular level and have yielded valuable details on sources of morphological variation essential for beak evolution.

Comparative analyses in disparate types of birds have found that some candidate genes are differentially expressed by NCM in patterns associated with species-specific variation in beak size and shape. For instance, Darwin’s finches, cockatiels, chicks, and ducks express *Bmp4* in their frontonasal primordia in well-defined domains that correlate with their distinct upper beak morphology, and experimentally expanding spatiotemporal expression of *Bmp4* in mesenchyme of chick embryos alters zones of proliferation and outgrowth of skeletal tissues and makes the beak broader and deeper (54, 117, 126, 146, 147). In this context, BMP signaling likely functions both as a species-generic osteo-inductive mechanism that supports skeletal outgrowth (101) and as a species-specific patterning mechanism whereby temporal and spatial expression of its members and targets in epithelium and mesenchyme are under the regulatory control of NCM (58, 87, 91).

Beyond testing for roles of known candidate genes, other studies have used high-throughput genome-based strategies to screen large numbers of genes concomitantly and have uncovered novel differentially expressed factors that associate with species-specific variation in beak morphology (16, 105, 117, 120). One landmark study combined a DNA microarray approach with tissues harvested from Darwin’s finches on the Galápagos Islands and found that the protein calmodulin, which mediates cellular calcium signaling, is expressed at elevated levels in finch species with long and pointed beaks (2). Moreover, experimentally upregulating calmodulin-dependent signaling in frontonasal mesenchyme of chick embryos elongates the upper beak skeleton. Other genes, including *Alx1*, which is expressed in NCM (96), are also associated with beak shape variation in Darwin’s finches (75). Such results add further support to the hypothesis that NCM-mediated spatial domains and levels of gene expression are fundamental to controlling species-specific size and shape in the beak.

To test the extent to which NCM establishes species-specific size and shape of the beak by controlling patterns of gene expression and to identify regulatory mechanisms that these cells employ to do so, we developed the quail–duck chimeric transplant system (46, 85, 121). Transplanting presumptive NCM destined to form the beak between quail and duck provides a relatively clear-cut way to identify donor-versus host-mediated patterning mechanisms (Figure 2e). The fact that quail mature on a highly accelerated timetable relative to duck allows us to readily screen chimeric embryos for donor-induced changes to the timing of gene expression, tissue differentiation, and/or other events during development. Also, donor and host contributions can be distinguished from one another perpetually by applying an antibody that recognizes cells from quail but not duck (121) or by using a polymerase chain reaction–based strategy that relies on genetic polymorphisms between these species (32). Thus, the quail–duck chimeric system can sort out the roles of NCM during beak development and identify molecular mechanisms that generate species-specific pattern.

In our initial transplant experiments, we observed that quail NCM generated short, blunt quail-like beaks on duck hosts (which we call quck chimeras), whereas duck NCM gave rise to long, broad duck-like bills on quail hosts (i.e., duail chimeras) (121). Equivalent transformations were observed in a parallel series of experiments where NCM destined to form the jaw joint was transplanted from quail to duck embryos and resulting chimeras acquired quail donor morphology (139). A pivotal insight about the role of NCM was gained by examining changes to beak tissues derived from the host. At the distal tip of their upper bill, duck normally form an egg tooth resembling a flat epidermal nail, while quail have an egg tooth that is more like a cone-shaped protrusion of hard keratin (84). The quck egg tooth, although derived solely from nontransplanted duck host epidermis, looked like that observed in quail (Figure 2f), and in reciprocal transplants, the duail egg tooth became duck-like in response to duck donor NCM (121). Such results demonstrate that species-specific patterning information is transferred from NCM to non-NCM-derived tissues, such as the rhamphotheca, and point to how NCM likely bolsters coevolution of different beak tissues as an integrated morphological unit (116, 120).

While the ability of NCM to confer species-specific pattern in the craniofacial complex had been known for more than 65 years (120), a foremost discovery from our work was finding that donor NCM implements species-specific pattern by executing autonomous molecular programs (e.g., transcription factors) in beak primordia and by regulating gene expression (e.g., signaling molecules) in adjacent host epithelium (121). This, in turn, guides differentiation and three-dimensional growth of tissues associated with the beak and ultimately fosters integration of form and function during development and evolution (144). For example, we have demonstrated that in quck chimeras, quail donor NCM causes duck host mesoderm-derived jaw adductor muscles to elongate rostrally and attach dorsally as in quail instead of proximally and laterally as in duck (131, 137). This repatterning of the shape, orientation, and insertion of mandibular adductor muscles is preceded by changes in spatial and temporal patterns of gene expression for muscle connective tissue markers in quail donor NCM, including *Tcf7l2* (120), which is a transcription factor that functions downstream of WNT signaling and plays a critical role in presaging muscle pattern (70). These NCM-mediated changes alter the functional morphology and associated mechanical force environment such that the quail donor side of quck chimeras fails to express mechanically responsive genes or form the secondary cartilage normally found at the insertion of mandibular adductor muscles on the coronoid process, whereas the control duck host side continues to produce secondary cartilage (131, 143). Thus, NCM-derived muscle connective tissues play a critical role in patterning muscles that enable species-specific beak movements.

Our quail–duck chimera studies have also shown that NCM conveys stage-specific and species-specific size and shape to the beak skeleton by regulating spatial and temporal deployment of molecular and cellular programs underlying each step of chondrogenesis and osteogenesis, including the induction, differentiation, and deposition of cartilage and bone (119). While chondrogenic ligands such as those from the FGF pathway are expressed broadly and continuously in epithelia surrounding the developing beak primordia, the timing of signal transduction and initiation of chondrogenesis itself are determined by when and where NCM first begins to express FGF receptors, including *Fgfr2* (33, 131, 143). The same holds true for osteogenic induction by BMP and TGFβ signaling and the subsequent formation of bone via expression of downstream effectors such as *Runx2* (58, 91, 130).

Another notable insight we made is that developmental timing plays a critical role in establishing beak size and that NCM dictates when bone forms in the beak by controlling cell cycle progression through stage- and species-specific expression of cell cycle regulators (58). Changes to the timing of developmental events (i.e., heterochrony) have long been postulated as a mechanism contributing to morphological evolution (119). Our studies suggest that differences in expression or posttranslational processing of cyclin and cyclin-dependent kinase (CDK) inhibitors, for example, may allow quail to reduce the proliferation of NCM and form a faster-developing and ultimately smaller beak. We find that the CDK inhibitor p27 is upregulated in quail and chimeric quck relative to duck (58), which is also consistent with previous studies showing that p27 expression is lower in the developing frontonasal process of duck relative to chick (105).

Additional understanding of mechanisms that link cell cycle progression, cell proliferation, osteogenic differentiation, and beak size come from our studies on the transcription factor *Runx2*. As a master regulator, *Runx2* responds to and modulates cell cycle genes by repressing ribosomal RNA (rRNA) synthesis and upregulating p27 expression (119). *Runx2* is a target of the TGFβ and BMP pathways, is expressed by osteogenic populations of NCM, is substantially elevated in beak primordia of quail versus duck, and is expressed prematurely and at higher quail-like levels in quck chimeras (58, 91). Inducing early exit from the cell cycle mimics the accelerated and higher levels of *Runx2* expression observed in chimeras. Furthermore, prematurely overexpressing *Runx2* in chick embryos decreases beak size to be more like that found in quail (34, 58).

Taken together, our work on *Runx2* suggests that NCM controls beak size by maintaining intrinsic species-specific levels of *Runx2*, which in turn affects the timing of bone formation. Such results also lend support to studies that have proposed a mechanistic link between predicted levels of *Runx2* expression [based on ratios of tandem repeats in glutamine (Q) and alanine (A)] and facial length in dogs and other mammals (95). NCM of duck embryos proliferates more slowly, and the population expands over longer periods of absolute time before undergoing differentiation, which ultimately leads to larger skeletal elements in the beak. By contrast, NCM of quail embryos represses proliferative signals sooner, exits the cell cycle earlier, and generates a relatively smaller beak size. This species-specific shift in the timing of the transition from proliferation to differentiation likely affects the size, shape, and location of mesenchymal condensations, which is a critical phase of skeletogenesis that can serve as a developmental mechanism for morphological evolution (57, 129).

The quail–duck chimeric system has also led us to discover another fundamental developmental mechanism contributing to beak size and shape. By analyzing late embryonic stages of beak outgrowth in quail and duck, we have found significantly higher levels and distinct spatial domains of markers for bone resorption in the quail beak skeleton (Figure 2g). By contrast, the developing duck bill shows little evidence of bone resorption in zones that would restrict its lengthening along the anterior–posterior axis (31) (Figure 2h). Quck chimeras validate that quail donor NCM upregulates markers of bone resorption (Figure 2i), especially the expression of *Matrix metalloproteinase 13* (*Mmp13*), which is many times higher in quail and chimeras at key developmental stages (31). When we experimentally inhibit bone resorption or MMP13 activity in quail embryos, we can lengthen the beak, whereas if we activate bone resorption, we can shorten the beak. Such results have also been confirmed by inhibition of additional factors such as *Mmp9* and *Cathepsin K* that regulate bone resorption in the beak (64). Thus, beak size appears to be inversely proportional to levels of bone resorption, and such levels are set by NCM. In this capacity, NCM seemingly acts as a rheostat during beak evolution that can be turned up or down to create local zones of resorption that sculpt bone by inhibiting or promoting directional growth (120). This finding ties in with studies on beak length and calmodulin signaling, which is also known to regulate bone resorption (117). What have remained elusive, however, are the precise regulatory mechanisms through which NCM differentially controls gene expression in a species-specific manner. Moreover, additional relevant genes remain to be discovered. Important clues in this regard have been unearthed by comparing sequences of individual candidate genes and whole genomes across divergent bird taxa, as well as GWAS that focus on beak size and shape.

## 5. GENETIC AND EPIGENETIC MECHANISMS OF BEAK PATTERNING

Our studies on the crucial role of bone resorption in mediating beak length led us to investigate genetic mechanisms that could account for how quail differentially express substantially higher levels of bone-resorbing enzymes such as *Mmp13*. We focused on mandibular primordia and found that quail have greater activation of and sensitivity to TGFβ signaling than do duck, where mediators such as SMADs and targets such as *Runx2*, which bind *Mmp13*, are more elevated (130). Inhibiting TGFβ signaling decreased bone resorption, and overexpressing *Mmp13* in NCM shortened the duck lower jaw. To understand the genetic basis for this differential regulation we examined the *Mmp13* promoter and discovered a SMAD-binding element and single-nucleotide polymorphisms (SNPs) near a RUNX2-binding element that distinguished quail from duck within the first 184 bases upstream of the translation inititation site (Figure 2j). When we removed the SMAD site and switched the species-specific SNPs, we abolished TGFβ sensitivity in the quail *Mmp13* promoter, but adding a SMAD site and quail/chick SNPs made the duck promoter responsive. Thus, we demonstrated that differential regulation of TGFβ signaling and *Mmp13* promoter structure mediate *Mmp13* expression and bone resorption and ultimately contribute to species-specific variation in lower beak length during development and evolution. TGFβ signaling alongside WNT signaling has also been implicated in differentially affecting the premaxillary component of the upper beak in Darwin’s finches (86).

Regulatory changes such as the ones we uncovered in the *Mmp13* promoter have been viewed as a central mechanism for evolutionary diversification as opposed to modifications to coding sequences of genes (130). Many studies have proposed a role for *cis*-regulatory enhancers, and deciphering the morphogenetic consequences of mutations in these regulatory domains has helped illuminate indispensable mechanisms of development, disease, and evolution. For example, regulatory changes in genes expressed by NCM have driven craniofacial evolution on a microevolutionary timescale (12), and mutations in noncoding regions and other loci also appear highly correlated with evolutionary changes in beak morphology across major clades of birds as well as within groups such as Darwin’s finches, Hawaiian honeycreepers, and various species of tits (Passeriformes) (18, 21, 54, 75, 111, 145, 152). One longitudinal study of great tits (*Parus major*) in the United Kingdom and Netherlands found that genomic regions under differential selection contained genes positively correlated with beak length, including *Collagen type IV alpha 5* (10). Many other genes in birds, particularly ones related to bone deposition and resorption as well as edentulism, show evidence of positive selection and/or pseudogenization (153).

Comparative genomic strategies continue to identify candidate genes and/or regions of the genome that are under natural selection and correlate with evolutionary variation in beak size and shape. As would be expected, such strategies have substantiated that numerous genes (and evidently their associated noncoding regions) affect beak morphology, that these genes are distributed widely throughout the genome, and that nearly all genetic changes appear to be species-specific, although some of the same signaling pathways and large-effect genes seem to be shared by many different bird taxa (145). While most of these candidate genes remain to be tested experimentally for the extent to which they play a role during beak morphogenesis, strong correlations of beak genotype to phenotype continue to emerge from data sets incorporating comparative genomics with phylogenetic systematics and geometric morphometrics (9, 152). As sequencing and bioinformatic technologies advance, all 10,000 extant lineages of birds [and likely some extinct ones (25)] may eventually have their genomes characterized comprehensively (43, 133, 145). Such an achievement would undoubtedly encourage rapid discovery of novel factors regulating avian beak development and evolution.

In addition to mutations in coding and noncoding regions of the genome, other genetic and epigenetic mechanisms are constantly being discovered that affect beak size and shape. For example, hundreds of microRNAs, which are small noncoding molecules that regulate gene expression, are differentially expressed in frontonasal primordia among duck, chick, and quail intriguingly at embryonic stages when species-specific differences in growth trajectories are observed (105). But functions for most of these microRNAs remain unknown. Likewise, epigenetic mechanisms such as DNA methylation distinguish rural and urban populations of Darwin’s finches and associate with beak size differences and genes in the BMP and TGFβ pathways (73, 90). These and other epigenetic mechanisms including bivalent chromatin at promoters of critical genes may boost the ability of NCM to adapt rapidly to changes in the local developmental environment, which in turn could generate variation in beak morphology among individuals or even species (123).

Regardless of whether mechanisms involve changes in genome organization, *cis*-regulation of individual genes, transcriptional and posttranscriptional regulation at the epigenetic level, biochemical interactions among gene products such as enzymes and other proteins, posttranslational modification of proteins, diffusion-reaction gradients and thresholds that affect inductive capacities of cells, movements and properties of cells, and/or physical and signaling interactions between tissues, the downstream implications for beak morphology can be profound (119). Modulating developmental programs at any of these hierarchical levels of organization across various lineages of birds could generate the phenotypic variation required for beak evolution, but, at the same time, such variation is also constrained by seemingly stable networks of genes and robust internal mechanisms that maintain conservation in beak morphology among all birds (144).

## 6. CONCLUSIONS AND FUTURE DIRECTIONS

Strikingly, almost all morphological variation in avian beaks arises in those structures either derived from NCM or patterned by NCM. Such a conclusion inspires the question, What endows this embryonic population of cells with the plasticity to drive evolutionary diversification? The unique capacity of NCM to function at the upper echelon of hierarchies through which developmental programs and gene regulatory networks convey species-specific pattern is a likely explanation (120). Given its contributions and patterning role across multiple systems, including the nervous, neuroendocrine, integumentary, and musculoskeletal, NCM and the regulatory changes therein can serve as a major mechanism for coevolution of form and function (112, 144), which in birds includes beak size and shape, biomechanics, and behavior.

Ultimately, beak pattern in any given species is established by the differential expression of its genome in three dimensions over absolute and/or relative time (119). In this framework, an ongoing question for current and future research involves identifying at a systems level precisely where, when, and how variation in genetic, molecular, and cellular programs directed by NCM generates species-specific changes in beak size and shape during development and evolution. Answering this question will likely require large comparative data sets (e.g., genomic, transcriptomic, and proteomic) across numerous species, sophisticated methods for computational analyses and the visualization of results, and robust hypothesis testing using state-of-the art tools in experimental embryology, including large-scale editing and re-engineering of the genome.
